# *CSMD1* Shows Complex Patterns of Somatic Copy Number Alterations and Expressions of mRNAs and Target Micro RNAs in Esophageal Squamous Cell Carcinoma

**DOI:** 10.3390/cancers14205001

**Published:** 2022-10-13

**Authors:** Nan Hu, Chaoyu Wang, Tongwu Zhang, Hua Su, Huaitian Liu, Howard H. Yang, Carol Giffen, Ying Hu, Philip R. Taylor, Alisa M. Goldstein

**Affiliations:** 1 Division of Cancer Epidemiology and Genetics (DCEG), National Cancer Institute (NCI), Bethesda, MD 20892, USA; 2 Center for Cancer Research (CCR), National Cancer Institute (NCI), Bethesda, MD 20892, USA; 3 Information Management Services, Inc., Silver Spring, Bethesda, MD 20904, USA; 4 Computational Genomics & Bioinformatics Branch (CGBB), Center for Biomedical Informatics and Information Technology (CBIIT), National Cancer Institute (NCI), Bethesda, MD 20892, USA

**Keywords:** esophageal squamous cell carcinoma, *CSMD1*, somatic copy number alternation, allelic imbalance, gene expression, miRNA

## Abstract

**Simple Summary:**

Human Cub and Sushi Multiple Domains 1 (*CSMD1*) is a novel candidate tumor-suppressor gene. We investigated *CSMD1* in esophageal squamous cell carcinoma (ESCC) by performing an integrated analysis of somatic DNA alterations (i.e., copy number alteration, allelic imbalance, and loss of heterozygosity) with RNA expressions (mRNA and target miRNAs) on specimens from the same ESCC patients, using data from SNP, miRNA, and RT-PCR arrays. Our results indicate that the *CSMD1* gene may play a role in the development of ESCC through complex patterns involving somatic alterations and mRNA expression. Furthermore, somatic copy number alterations in SNPs located in non-coding regions of *CSMD1* appear to influence expression of both this gene and its target miRNAs.

**Abstract:**

Background: Human Cub and Sushi Multiple Domains 1 (CSMD1) is a novel candidate tumor-suppressor gene that codes for multiple domains, including complement regulatory and adhesion proteins, and has recently been shown to have alterations in multiple cancers. We investigated CSMD1 in esophageal squamous cell carcinoma (ESCC) by performing an integrated analysis on somatic copy number alterations (CNAs), including copy-number gain or loss, allelic imbalance (AI), loss of heterozygosity (LOH), and the expressions of mRNA and its target miRNAs on specimens from the same patients with ESCC. Results: (i) Two-thirds of ESCC patients had all three types of alterations studied—somatic DNA alterations in 70%, and abnormal expressions of CSMD1 RNA in 69% and in target miRNAs in 66%; patterns among these alterations were complex. (ii) In total, 97% of 888 CSMD1 SNPs studied showed somatic DNA alterations, with most located near exons 4–11, 24–25, 39–40, 55–56, and 69–70. (iii) In total, 68% of SNPs with a CNA were correlated with expression of CSMD1. (iv) A total of 33 correlations between non-coding SNPs and expression of CSMD1 target miRs were found. Conclusions: Our results indicate that the *CSMD1* gene may play a role in ESCC through complex patterns of DNA alterations and RNA and miRNA expressions. Alterations in some somatic SNPs in non-coding regions of *CSMD1* appear to influence expression of this gene and its target miRNAs.

## 1. Introduction

The human Cub and Sushi Multiple Domains 1 (*CSMD1*) gene is thought to be a novel candidate tumor-suppressor gene that codes for multiple domains, including complement regulatory adhesion proteins and a membrane protein with an extracellular region, a single transmembrane domain, and a short cytoplasmic domain, although its function in human tumors is currently unknown [1,2]. *CSMD1* consists of 70 exons, spans two megabases in chromosomal region 8p23.2 (chr8: 2,935,353–4,994,806, hg19), and encodes an 11.5 kilobase transcript [1]. It is composed of CUB and complement control protein (CCP) domains and therefore shares homology with several proteins involved in many cellular processes such as growth, cell adhesion, cancer progression, and control of the complement system [3]. Studies on *CSMD1* show that it is expressed abundantly in the central nervous system and at intermediate levels in normal human oral and oropharyngeal epithelia [4]. *CSMD1* has also been studied in other cancers, and decreased expression was found in lung, head and neck, breast, skin, colorectal, gastric, and ovarian cancers [1,5,6,7,8,9]. Somatic mutation, allelic imbalance, and methylation in *CSMD1* were identified in colorectal adenocarcinoma and appeared to correlate with earlier clinical presentation [10,11]. Methylation of *CSMD1* was also shown in head and neck squamous cell carcinoma cell lines [12] and in childhood acute lymphoblastic leukemia [13]. Homozygous deletion of *CSMD1* with decreased expression was found in ovarian serous tumor, hepatocellular, and oral squamous cell carcinoma [9,14,15]. Kamal et al. reported that reduced CSMD1 protein expression was associated with high tumor grade and poor survival in invasive ductal breast carcinoma [2]. Studies have also investigated the mechanisms of *CSMD1* action [3,16]. For example, Tang et al. linked *CSMD1* with the Smad family in studies using melanoma cells and proposed a signaling mechanism for *CSMD1*-induced apoptosis [16]. In our previous studies, we found that 23% of esophageal squamous cell carcinoma (ESCC, 7/30) cases exhibited biallelic loss [17] and three of four ESCC cases had exonic somatic mutations in *CSMD1*, using whole-genome sequencing [18]. Furthermore, Gao et al. reported that somatic mutations in this gene occurred in 8% of 113 Chinese ESCC cases, using whole-exome sequencing [19]. *CSMD1* mutations in ESCC were also found by Moody et al. based on whole-genome sequencing conducted on ESCC patients from eight countries [20]. Taken together, these studies suggest that *CSMD1* may play an important role in the development of human tumors, but it has been little studied in ESCC.

MicroRNA (miRNA) is thought to be a potential contributor of regulatory non-coding RNAs. However, few studies of *CSMD1* RNA expression and its target miRNAs in human cancers have been reported. One previous study showed overexpression of miR-10a and miR-10b in glioblastoma stem cells and normal neural stem cells and both miR10a and miR-10b could repress *CSMD1* expression [21]. Another report also showed that miR-10b decreased *CSMD1* expression in human hepatocellular carcinoma cells [22]. We previously conducted an integrated analysis of genome-wide miRNA and gene expression in ESCC, and the results provided insights into the expression of miRNAs and their relation to regulation of RNA targets in ESCC tumorigenesis, suggesting opportunities for the future development of miRs and mRNAs as biomarkers for early detection, diagnosis, and prognosis in ESCC [23]. A separate evaluation of two different eQTL analyses indicated that SNPs in non-coding regions were not only potential risk biomarkers by classic analysis strategies, but also carried somatic CNA that might influence gene expression in ESCC and be identified by using an integrated analytic approach [24].

Esophageal cancer is the seventh most common and the sixth most fatal human cancer in the world [25]. In China, overall, it is the fourth leading cause of cancer mortality [26]. Shanxi Province, a region in North–Central China, has among the highest rates of ESCC in China. Since ESCC is a complex and heterogeneous disease which is typically diagnosed only after the onset of symptoms when prognosis is very poor, it is increasingly important to understand the molecular biology of ESCC so that new and better markers of early disease, as well as pathways amenable to targeted therapies, can be found to advance both early detection and molecular therapy strategies capable of reducing the excessive morbidity and mortality associated with this disease. Thus, in the present study, we studied somatic alterations at the DNA, mRNA, and miRNA level in ESCC to investigate the relation of DNA somatic changes to expression of mRNA and target miRNAs in *CSMD1* to help us better understand the role of SNPs in non-coding regions with somatic alterations in expression in this ESCC tumor gene.

## 2. Materials and Methods

### 2.1. Cases, Specimen Processing, and Arrays

#### 2.1.1. Case Selection

This study was approved by the Institutional Review Boards of the Shanxi Cancer Hospital and the US National Cancer Institute (NCI). Briefly, cases diagnosed with ESCC between 1998 and 2004 in the Shanxi Cancer Hospital in Taiyuan, Shanxi Province, PR China. Patients were from five geographic regions (Taiyuan, Linfen, Jinzhong, Chanzi, and Xinzhou), which cover two-thirds of Shanxi province. Patients who were considered candidates for curative surgical resection were identified and recruited to participate in this study after obtaining informed consent. None of the cases had prior therapy, and Shanxi was the ancestral home for all. Our analysis here was based on 56 ESCC cases who ranged in age from 39 to 71 years (median 57 years) and were predominantly male (64%). Around 30% (17/56) of cases had upper gastrointestinal (UGI) cancer in their family history. Clinically, most cases had Stage 2 (68%) cancers, and 55% (31/56) had evidence of lymph node metastasis at diagnosis. Survival time ranged from 1.1 months to 63.5 months (median 24.1 months) for 53 cases with known follow-up information. Three cases’ families moved away from Shanxi Province and were lost to follow-up (Supplementary Table S1).

#### 2.1.2. Biological Specimen Collection and Processing

Venous blood (10 mL) was taken from each case prior to surgery, and germline DNA from whole blood was extracted and purified by using the standard phenol/chloroform method and stored at −80 °C. Samples were shipped to the NCI on dry ice. Tumor and adjacent normal tissues were dissected at the time of surgery and stored in liquid nitrogen until use. DNA and total RNA were extracted from tumor by using the Allprep kit (Qiagen, Hilden, Germany) per the manufacturer’s instructions. The quality and quantity of both germline and tissue DNA were assessed by using the NanoDrop 2000 (Thermo Scientific, Waltham, MA, USA) for individual samples. The DNA quality was assured by only including samples with 260/280 ratios between 1.8 and 2.0. The RNA quality and quantity were determined by using the RNA 6000 Labchip/Agilent 2100 Bioanalyzer (Agilent Technologies, Germantown, MD, USA). The RNA quality was assured by only including samples with 28S/18S ratios between 1.9 and 2.1.

#### 2.1.3. Target Preparation for GeneChip Human Mapping 500K Array Set

The Affymetrix GeneChip Human Mapping 500K array and SNP 5.0 set were performed in these patients. The 500K array contains ~262,000 (Nsp I array) and ~238,000 (Sty I array) SNPs (mean probe spacing = 5.8 Kb, mean heterozygosity = 27%). SNP5.0 contains all 500,568 SNPs from the two-array Mapping 500K array set, as well as an additional 420,000 non-polymorphic probes. SNPs on the array are present on 200 to 1100 base pairs (bp). A detailed gene chip protocol can be found at http://www.affymetrix.com. Experiments were conducted according to the protocol (GeneChip Mapping Assay manual) supplied by Affymetrix, Inc. (Santa Clara, CA, USA). Genotype calls were generated by GTYPE v 4.0 software (Affymetrix, Santa Clara, CA, USA). Paired germline and tumor DNAs from each case were run together in parallel in the same experiment (i.e., same batch and same day). The GEO accession number for these SNP array data is GSE74705.

#### 2.1.4. *CSMD1* Gene Expression by Quantitative Reverse Transcription-PCR (qRT-PCR)

Reverse transcription of RNA was performed by adding 0.2 ug total RNA, 1 uL of oligo(dT)12–18 (500 ug/mL), 1 uL (200 units) of SuperScript II reverse transcriptase, 1 uL (2 units) of E-coli RNase, and 1 uL of 10 mmol/L deoxynucleotide triphosphate (Invitrogen) in total volume of 20 uL. All real-time PCRs were performed by using an ABI 7300 Sequence Detection System. Primers and probes of *CSMD1* gene and an internal control gene (*GAPDH*) were designed and ordered from ABI. A singleplex reaction mix was prepared according to the manufacturer’s protocol of “Assays-on-Demand Gene Expression Products”, including 10 uL Taqman Universal PCR Master Mix, No-AmpErase UNG (2X), 1 uL of 20X Assays on-Demand Gene Expression Assay Mix (all Gene Expression assays have a FAM reporter dye at the 5′ end of the Taqman MGB probe and a nonfluorescent quencher at the 3′ end of the probe), and 9 uL of cDNA (1000 ng) diluted in RNase-free water to a total volume of 20 uL. Each sample for the gene was run in triplicate. The thermal cycling conditions included an initial denaturation step at 95 °C for 10 min, 40 cycles at 95 °C for 15 s, and 60 °C for 1 min. The 11 selected probes covered exons 4–11, 24–25, 39–40, 55–56, and 69–70 (overall, covering 16 coding regions), based on the results of DNA somatic alterations. The selected probes were designed to span the junction between two exons (exome boundary). For example, the boundary exon 69–70 binding region for the probes is partially in exon 69 and partially in exon 70. The entirety of exons 69 and 70 was not targeted (Supplementary Table S2).

#### 2.1.5. ABI miRNA Expression Array by RT-PCR

The TaqMan^®^ Low Density Array was used to determine microRNA expression in this study, which employed the 9700HT fast real-time PCR system from ABI. Comprehensive coverage of Sanger miRBase v14 was enabled via a two-card set of TaqMan^®^ Array MicroRNA Cards (Cards A and B) for a total of 664 unique human miRNAs. The method was previously described [23]. The GEO accession number for these miRNA expression array data is GSE67269.

### 2.2. Data Analysis

#### 2.2.1. GeneChip 500K and SNP5 Array Data Analysis

Probe-intensity data from arrays of Affymetrix 500K and SNP 5 were used to identify somatic DNA alterations in the present study. To avoid gender-related issues, SNPs mapped to either the X or Y chromosome were excluded. Affymetrix SNP array data were first normalized by using the gtype-probe set-genotype package included in Affymetrix Power Tools version 1.85.

*CSMD1* on GeneChip 500K and SNP5 array data analysis using Nexus 6.1:

The raw data [CEL file] of 20 paired samples from Affymetrix 500k and 36 paired samples from Affymetrix 5.0 were loaded by using R/bioconductor and aroma.affymetrix. The aroma.affymetrix package can process nearly all Affymetrix Copy Number and SNP arrays and can provide estimates of total copy number for all loci and allele B fractions for all (bi-allelic) SNPs. For more details, go to http://www.aroma-project.org/. We used the CRMA function implemented in the Aroma to preprocess and applied the CBS method for segmentation. We combined the segmentation results from two platforms and imported the results to Nexus Copy Number 6.1, following the manual instructions (www.Biodiscovery.com). We used Q-Bound cutoff at 0.5 and G-Score cutoff at 1.0 to identify the focal regions of amplification and deletion with FDR q-value 0.25 for copy number, using GISTIC 2.0, as recommended.

For identifying physical location (exon or intron), an in-house python script was developed to retrieve the gene name and intron location for each SNP in our list from hg19 Refseq database from the UCSC genome browser (http://hgdownload.soe.ucsc.edu/goldenPath/hg19/database/ (accessed on 22 June 2022)). Transcripts with accessions starting with “NM_” were used. A total of 888 *CSMD1* SNPS were found to have the necessary information (physical location, introns/exons flank sequence, and alleles A and B) to be included in our analysis, and all are located in intronic regions of *CSMD1*.

The criteria for DNA segment alterations are as follows: (i) There was a significance threshold of 5.0 × 10^−6^, max contiguous probe spacing of 1000 kbp, and the minimum number of probes per segment of three. (ii) The copy number (CN) was defined by using the Log R ratio (LRR) as follows: CN high gain ≥ 1.0, CN gain > 0.2, CN big loss ≤ −1.0, and CN loss < −0.2. (iii) AI and LOH were identified by using B Allele Frequency (BAF) as follows: Allelic imbalance was identified when BAF was 0.2–0.45 or 0.55–0.80, and LOH was identified when BAF was <0.2 or >0.8.

#### 2.2.2. CSMD1 Gene Expression by qRT-PCR Data Analysis

Gene expression was analyzed by using the 2^−ΔΔCT^ algorithm. Details of the 2^−ΔΔCT^ method have previously been described [27] (http://docs.appliedbiosystems.com/search (accessed on 22 June 2022); [28]). Briefly, the mean target gene mRNA expression level for the three mRNA measurements was calculated. The 2^−ΔΔCT^ method was used to calculate relative changes in gene expression determined from real-time quantitative PCR experiments. In the present study, the data are presented as the fold change in each exon of *CSMD1* expression in tumors normalized to the internal control gene (*GAPDH*) and relative to the normal control (matched normal as calibrator). The results of the real-time PCR data were represented as C_T_ values, where C_T_ was defined as the threshold cycle number of PCR at which the amplified product was first detected. There is an inverse correlation between C_T_ and amount of target: lower amounts of target correspond to a higher C_T_ value, and higher amounts of target have lower C_T_ values. The average C_T_ was calculated for both the target genes and *GAPDH*, and the ΔC_T_ was determined as (the mean of the triplicate C_T_ values for the target gene) minus (the mean of the triplicate C_T_ values for *GAPDH*). The ΔΔC_T_ represented the difference between the paired tissue samples, as calculated by the formula ΔΔC_T_ = (ΔC_T_ of tumor − ΔC_T_ of normal). The N-fold differential expression in the target gene of a tumor sample compared to the normal counterpart was expressed as 2^−ΔΔCT^ [27] (http://docs.appliedbiosystems.com/search (accessed on 22 June 2022)) [28]. In the present study, the range of mRNA expression was defined by the N-fold change as overexpressed (N-fold change ≥ 2.0), normal (N-fold range from 0.5001 to 1.9999), or underexpressed (N-fold change under ≤ 0.5).

#### 2.2.3. CSMD1 Target miRNA Expression Array Analysis

RQ Manager integrated in software from ABI was used to normalize the entire signal generated. The expression level (as fold change) was calculated when both tumor and normal samples had signals in the assays, using DataAssist software v2.0 (Life Technologies, Carlsbad, CA, USA, http://www.lifetechnologies.com/about-life-technologies.html (accessed on 22 June 2022)). Signals for miRNA present either in tumor only or normal only were dropped from our analysis. The fold change was calculated by using the 2^−ΔΔCT^ method, as described previously [29]. The criteria used to call a miRNA dysregulated was a fold change ≥ 2 or ≤0.5. We used TargetScan (http://www.targetscan.org/) (Whitehead Institute for Biomedical Research, Cambridge, MA, USA) and Sanger miRBase (http://www.mirbase.org/) to identify conserved miRNAs in the 3′ UTR for *CSMD1*. A total of 22 miRNAs were analyzed in the present study.

#### 2.2.4. Correlation Analyses

To determine whether SNPs in the non-coding region affected mRNA expression or not, we performed Spearman correlation analyses between the signal value of each SNP with a somatic DNA alteration (Log RR for CNA) and the value (fold change) of each exon of *CSMD1* and each target miRNA. Moreover, we performed Spearman correlation analyses between the expressions (fold changes) for each exon of *CSMD1* with each of its target miRNAs. All analyses were conducted by using R software. If there were less than five cases showing signals on a miRNA or a probe of an exon of *CSMD1*, the miRNA or probes for an mRNA were excluded from the analysis. All analyses considered comparisons with *p*-values < 0.05 to be significant (i.e., nominal significance).

#### 2.2.5. Survival Analysis

We performed a survival analysis for the mRNA expression of each miRNA target and each exon of *CSMD1* by using the Kaplan–Meier Estimator in the survival analysis package in R software, which also included hazard ratio estimations from proportional hazards models. The patients were divided into two groups according to fold change: >1 fold change (FC) and ≤1 FC. A significant difference in survival status was detected by using the log-rank test and *p*-value < 0.05.

## 3. Results

**Figure 1:**
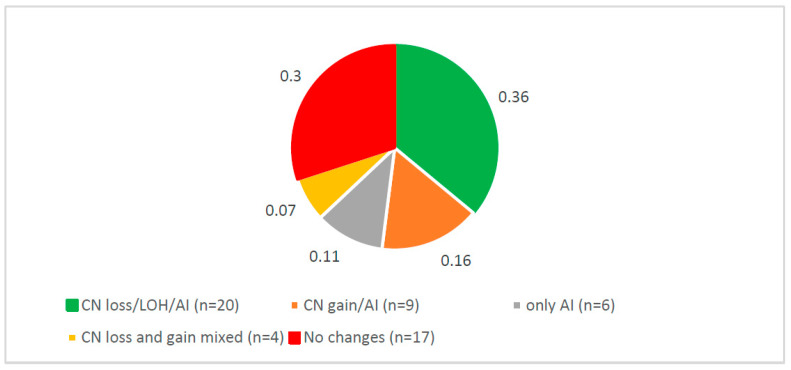
Distribution of somatic DNA alterations on *CSMD1* in 56 ESCC.

**Figure 2:**
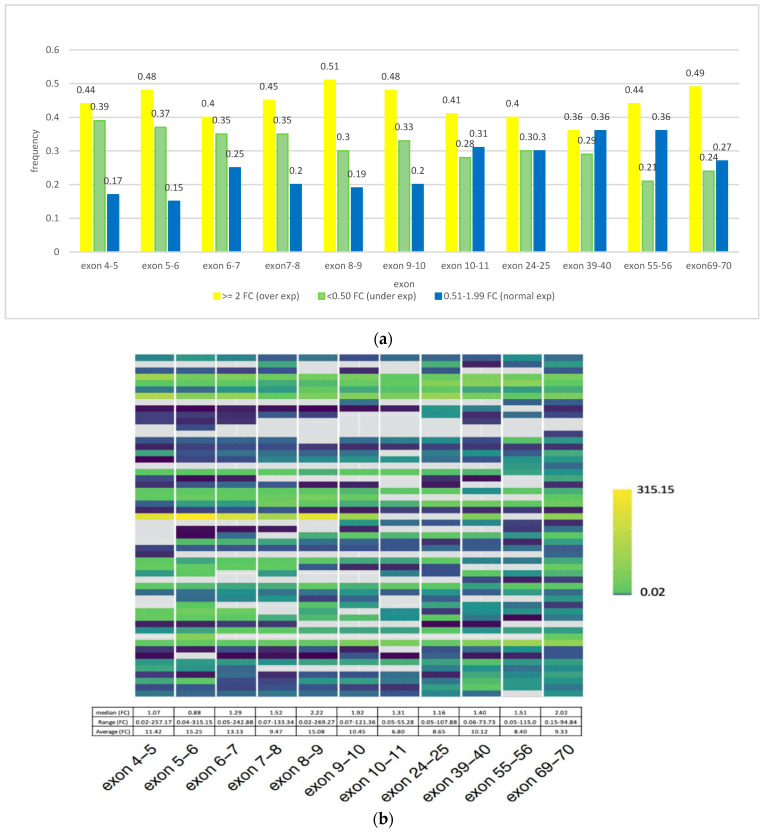
(**a**) Distribution of mRNA expression of *CSMD1* in ESCC. The yellow bar indicates the frequency of cases with overexpression (fold change (FC) ≥ 2.00); green bar indicates frequency of cases with underexpression (FC ≤ 0.50); blue bar indicates frequency of cases with expression in the normal range (FC 0.51–1.99). (**b**) Individual ESCC case mRNA expression levels for the 11 exon boundaries studied, shown as fold change (FC) (top, heatmap) and as median, range, and mean across the 54 cases examined in each of the 11 exons tested (bottom). Each row in the heatmap represents an individual case, and each column represents mRNA expression levels in a single exon boundary tested. FC ranges from lowest (0.02, blue) to highest (315.15, yellow). Gray denotes probe with no data available.

**Figure 3:**
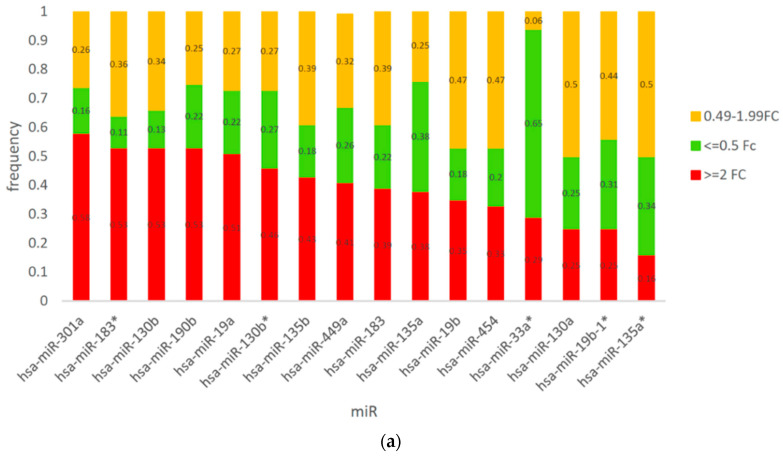

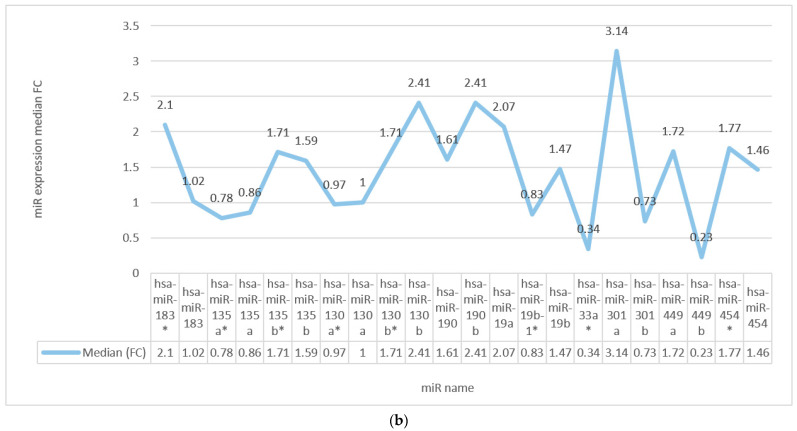
(**a**) Distribution of expression levels of *CSMD1* miRNA targets in ESCC cases. Red bars indicate miR overexpression (FC ≥ 2.00), green bars indicate miR underexpression (FC ≤ 0.50), and yellow bars indicate miR expression in the normal range (FC 0.49–1.99). (**b**) *CSMD1* target miR expression median fold change (FC) across 22 miRs, with the miRs shown across the horizontal axis.

**Figure 4:**
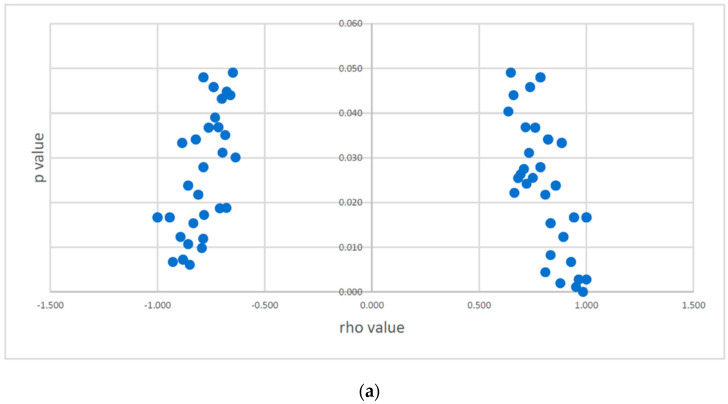

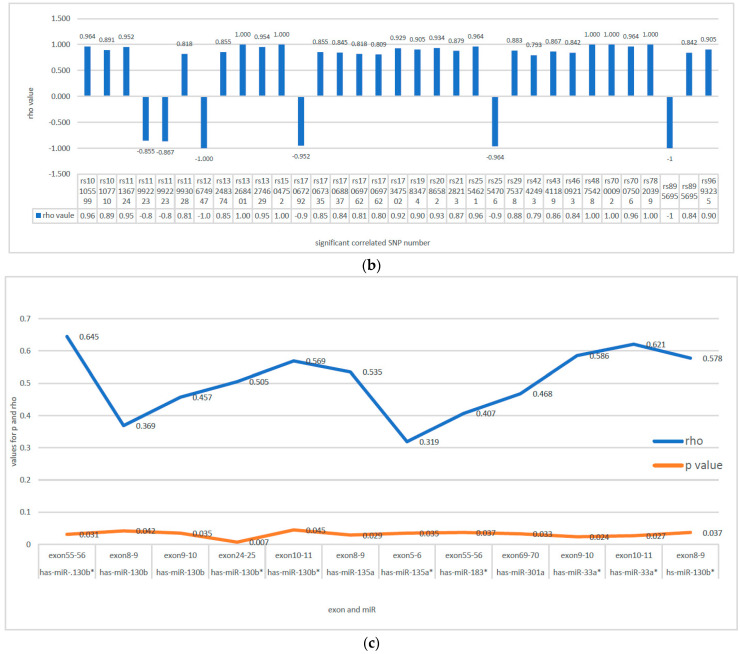
(**a**) Scatter chart of significant correlations between somatic CNAs in SNPs and mRNA expression of *CSMD1* in ESCC. Negative rho values on left side indicate inverse correlations; positive rho values on right side indicate positive correlations. (**b**) Significant correlations between SNPs with alterations and miR expression (*p* < 0.05). The y-axis is rho value, and the x-axis is the significantly correlated SNP. Each blue bar presents one correlation. (**c**) Significant correlations between expressions of *CSMD1* and its target miRNAs in ESCC. The x-axis shows exon number and target miR number; y-axis shows rho value (blue line) and *p*-value (orange line).

**Figure 5:**
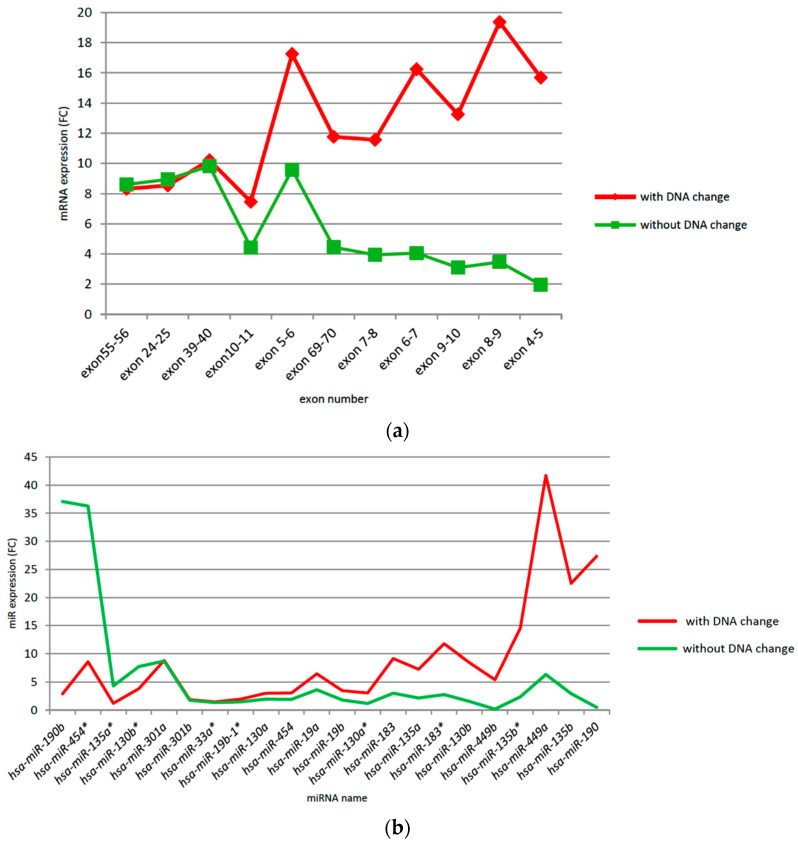
(**a**) *CSMD1* expression in ESCC with and without somatic copy number alterations, allelic imbalance, and LOH. (**b**) Expression of *CSMD1* target miRNAs in ESCC with and without somatic copy number alterations, allelic imbalance, and LOH.

**Figure 6:**
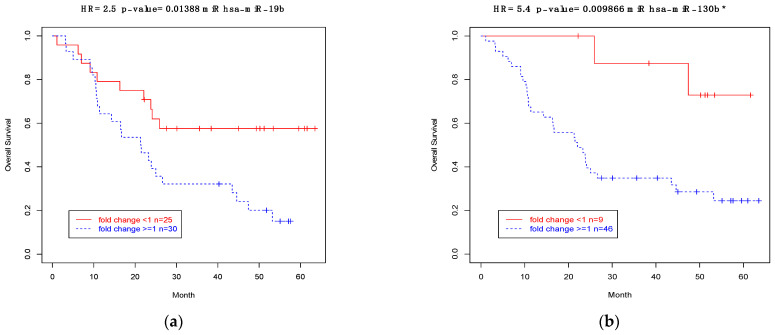
(**a**) ESCC case survival by miR-19b expression (Kaplan–Meier plot, HR from proportional hazards model). (**b**) ESCC case survival by miR-130b* expression (Kaplan–Meier plot, HR from proportional hazards model).

Overall, in the present study, we first assessed copy number-alterations (CNAs) in *CSMD1* as CNAs, AI, and LOH and determined that 70% of cases (39/56) had such DNA alterations (Figure 1). Second, we determined the frequency distribution of *CSMD1* mRNA expression for each case by category of expression (overexpression, underexpression, and normal expression) and showed that overexpression was the predominant category (Figure 2a). Individual-case mRNA expression levels for the 11 exon boundaries studied provided evidence for heterogeneity within individuals, as well as across probes (Figure 2b). Third, we examined the frequency distribution of expression levels of *CSMD1* miRNA targets in ESCC cases by categories (overexpression, underexpression, and normal expression) (Figure 3a), and also for each of the 22 separate miR expression levels in cases individually (Figure 3b). Table 1 shows the three alterations of *CSMD1* (DNA alterations and expression as fold changes for both mRNA and target miRs) in the 56 ESCCs. Finally, we examined the relations among the three alterations (Figure 4a–c and Figure 5a,b), which suggested that SNPs with somatic alterations from non-coding regions could influence expression of *CSMD1* RNA and its target miRs. Moreover, we showed associations between expressions of *CSMD1* and target miRNAs and survival in ESCC (Figure 6).

### 3.1. Complex Somatic DNA Alterations of CSMD1 in ESCC

A total of 888 SNPs in *CSMD1* were examined in the present study, all located in non-coding regions. In brief, 97% of these SNPs (864/888 SNPs) showed somatic CNAs, with most alterations around exons 4–11, 24–25, 39–40, 55–56, and 69–70 (involved 16 total coding regions) of *CSMD1*. These somatic CNAs showed complex patterns, suggesting ESCC heterogeneity. For example, case E0844 and case E1293 showed the CN gain on the entire gene, while case E1210 and case E1242 showed CN loss throughout *CSMD1*. The distribution of DNA alterations in each of the 56 ESCC cases is summarized in Table 1 and Supplementary Table S3. Figure 1 shows the distribution of somatic DNA alterations in *CSMD1* in the 56 ESCC patients. Overall, 70% of patients (39/56) showed somatic alterations, including CN loss/LOH/AI (36%), CN gain/AI (16%), and only AI (11%). ESCC patients could be divided into two groups based on the somatic alterations: 70% of patients (39/56) had somatic CNAs, while 30% (17/56) had no CNAs on *CSMD1* (Table 1). Among the 39 cases with somatic alterations, 20 had CN loss/LOH, including 15 with AI; 9 cases had CN gain, including 7 with AI; 6 cases had AI only; and 4 cases had mixed CN gain and loss (Figure 1). For example, case E1782 had a CN loss region on exons 3–70 and AI (Supplementary Figure S1a); case E1635 had CN gain for almost the entire gene (Supplementary Figure S1b); and case E1575 had CN gain covering exons 2–4 and CN loss covering exons 6–70 (Supplementary Figure S1c). Taken together, these results suggest that *CSMD1* has complex patterns for CNAs and AI in ESCC that are indicative of molecular heterogeneity in ESCC. If confirmed, this phenomenon may help identify ESCC subgroups that inform future studies of etiology, tumor biology, and prognosis/therapy.

### 3.2. CSMD1 mRNA Expression Using qRT-PCR and Its Relation to Somatic DNA Alterations in ESCC Cases

We selected specific exons in *CSMD1* to examine RNA expression based on the DNA alteration regions mentioned above. Eleven probes involving 16 regions (Supplementary Table S2) were used in the 54 available ESCC cases. More cases showed overexpression of *CSMD1* than normal or underexpression across the 11 probes evaluated (overexpression median frequency 0.44, range 0.36–0.51; underexpression median frequency 0.30, range 0.21–0.39; and normal expression median frequency 0.26, range 0.17–0.36; Figure 2a and Supplementary Table S4). Individual ESCC case mRNA expression levels for the 11 exon boundaries studied showed wide variation both across probes within individuals and across individuals by probe, as shown in the Figure 2b heat map, with an overall range for the data displayed of fold changes varying from 0.02 to 315 (Figure 2b and Supplementary Table S4). Median FCs for most exons were in the normal range, with the exception of exons 8–9 (FC 2.2) and exons 69–70 (FC 2.02), which both showed overexpression (Figure 2b and Supplementary Table S4). Sixty-nine percent (37/54) of cases showed abnormal expression of *CSMD1*, including 13 underexpressed (average FC ≤ 0.5) and 24 overexpressed (average FC ≥ 2). We also noted that patients with and without somatic DNA alterations showed similar percentages with abnormal expression of *CSMD1* 70% (26/37) and 65% (11/17), respectively.

By looking at individual cases, we observed several phenomena in relating mRNA expression to DNA alterations: (i) A subset of cases (8/26 = 31%) with CNAs/AI/LOH followed the same direction as their mRNA expression, while other cases showed opposite patterns (Supplementary Tables S3 and S4). (ii) We found that expression levels in various exons diverged within the same case. For example, in case E0387 (with CN loss and AI) two probes showed underexpression (FC 0.32 for exons 7–8 and 0.35 for exons 6–7; see Supplementary Figure S1d), two probes showed expression in the normal range (for exons 10–11 and 24–25), and six probes showed overexpression (FC from 2.20 to 9.16) in other exons. The expression range was from FC 0.32 (for exons 7–8) to 9.16 (for exons 5–6), which resulted in an average case FC of 2.77 for *CSMD1* gene expression. (iii) The status for cases with mixed CN gain/loss and LOH and their mRNA expression on *CSMD1* was also complex. For example, case E0410 had CN gain from exons 24 to 70 and LOH in the same region (Supplementary Figure S1e), while mRNA expression ranged from FC 0.10 (exons 5–6) to 3.27 (exons 69–70), which resulted in an average mRNA expression in the normal range (FC 1.05) (Supplementary Tables S3 and S4).

### 3.3. Expression of CSMD1 in Targeted miRNAs in ESCC

We used TargetScan (http://www.targetscan.org/) (Whitehead Institute for Biomedical Research, Cambridge, MA, USA) and Sanger miRBase (http://www.mirbase.org/) to identify 22 conserved miRNAs in the 3′ UTR for *CSMD1*, which are thought to be preferentially conserved. Supplementary Table S5 shows the expression levels across each of the 56 cases and the average FC, median FC, and range across each of the 22 miRNAs. Figure 3a shows the distribution of expression levels of each of these miRs in ESCC. Figure 3b shows the median expression FC (range 0.23–3.14) of the 22 miRNAs examined across all cases. Only seven miRs were considered abnormal, including five overexpressed miRNAs (≥2 FC; miR-183*, miR-130b, miR-190b, miR-19a, and miR-301a) and two underexpressed miRNAs (≤0.5 FC; miR-33a* and miR-449b) (Supplementary Table S5).

Sixty-six percent of cases (37/56) had abnormal expression of *CSMD1* target miRNAs based on average FC of all miRNAs tested. In these 37 cases, *CSMD1* was overexpressed in 36 (≥2 FC) and underexpressed in just 1 (≤0.5 FC). Similar abnormal expression rates of *CSMD1* target miRNAs were observed both for patients with (67%) and without DNA alterations (65%).

### 3.4. Correlation between SNPs with CNAs and Expression of CSMD1 and Its Target miRNAs in ESCC

#### Correlation between SNPs with CNAs and Expression of *CSMD1*

Among the 888 total SNPs examined, correlations were evaluated between each of the 123 SNPs with somatic CNAs in non-coding regions and the 11 miR probes. Although none of these comparisons reached the significance threshold after adjustment for multiple comparisons (3.69 × 10^−5^), 179 were nominally significant (*p* < 0.05) (Supplementary Table S6a). These 179 correlations included 58% (104/179) where somatic CNAs were positively correlated with *CSMD1* expression (either CN gain with overexpression, or CN loss with underexpression of *CSMD1*) with rho values ranging from 0.64 to 1.00, and 42% that were negatively (inversely) correlated, with rho ranging from −1.00 to −0.64 (Supplementary Table S6a; Figure 4a). Among 38 SNPs that correlated with multiple regions in *CSMD1*, 37 showed similar directionality with *CSMD1* expression; just one SNP (rs11784668) correlated in oppositive directions at different regions (exons 5–6 and 6–7 were positive, while exons 10–11 were negatively correlated). These results suggest that some SNPs with somatic CNAs located in introns are capable of influencing gene expression, either directly or indirectly, although the mechanisms are currently unclear.

### 3.5. Correlation between SNPs with CNAs and Expression of CSMD1 Target miRNAs

We performed correlation analyses between the 864 SNPs with somatic CNAs and the 19 miR probes that showed abnormal expression levels (average expression ≥2 FC or ≤0.5 FC). Thirty-three correlations between 30 SNPs with CNAs and expressions of 10 *CSMD1* targeted miRNA were nominally significant (*p* < 0.05). These included 27 positively correlated SNPs with rho values from 0.79 to 1.0, and 6 inversely correlated SNPs with rho values from −0.86 to −1.0 (Figure 4b). Of note, among the 33 correlated SNP alterations, 9 were with miR-135b, and 5 were with miR135a* (Supplementary Table S6b).

### 3.6. Correlation between Expression of CSMD1 mRNA and Target miRNAs

The expressions of seven *CSMD1* target miRNAs were significantly positively associated with expressions of exons 5–6, 8–9, 9–10, 10–11, 24–25, 55–56, and 69–70 (*p* < 0.05, rho range from 0.31 to 0.64) (Figure 4c). Three of the seven miRs were significantly correlated with more than one exon including has-miR-33a, has-miR-130b, and has-miR-130b*. Specifically, the expression of miR-130b*a was positively correlated with expression of exons 8–9, 10–11, 24–25, and 55–56. Moreover, miR-130b, miR-130b*, and miR135a expressions were positively correlated with expression of exons 8–9 (Figure 4c and Supplementary Table S6c).

We also identified seven SNPs that were significantly correlated with selected regions of *CSMD1* expressions as well as selected miRNA expressions (Table 2). For example, rs17068837 was negatively correlated with expression of exons 10–11 and positively correlated with expression of miR-135b.

### 3.7. CSMD1 Expression and Target miRNAs in ESCC Patients with and without Somatic DNA Alterations

ESCC cases with DNA alterations had higher expression levels of *CSMD1* than those without such alterations in most of the exons (9 of 11 boundaries), especially at exons 4–5 (average FC 15.69 vs. 1.95, or 8× higher) and exons 8–9 (average FC 19.37 vs. 3.47, or 5.6× higher) (Supplementary Table S7a and Figure 5a). A total of 16 of the 22 *CSMD1* target miRNAs showed higher expression levels in patients with DNA alterations than those without (e.g., miR-190 (average FC 27.34 vs. 0.48, or 57× higher), miR-135b (average FC 22.5 vs. 2.93, or 7.7× higher), miR-449a (average FC 41.71 vs. 6.31, or 6.6× higher), and miR-449b (average FC 5.4 vs. 0.17, or 6.9× higher)). In contrast, four miRNAs were expressed at lower levels in patients with DNA alterations than those without (e.g., miR-190b (average FC 2.9 vs. 37.07, or 0.08× lower)) (Supplementary Table S7b; Figure 5b). These results suggest that somatic DNA alterations may affect expression levels of *CSMD1* and target miRNA in ESCC, even when the somatic DNA alterations are in non-coding regions.

### 3.8. Risk Factors and Clinical Factors and Somatic DNA Alterations in CSMD1

The study unexpectedly showed that patients without a family history of upper gastrointestinal tract cancer had a significantly higher frequency of somatic alterations than those with a positive family history (79% vs. 47%, *p* = 0.026). Frequencies of somatic DNA alterations were similar between patients with (0.56) and without (0.53) lymph node metastasis.

### 3.9. Association between Expressions of CSMD1 and Target miRNAs and Survival in ESCC

Finally, we examined expression levels of *CSMD1* mRNA and its target miRNAs with patients’ survival by using expression fold changes, using Kaplan–Meier analyses. For mRNA expression, only the probe for exons 4–5 was nominally significantly correlated with patients’ survival (*p* = 0.047). Only 2 of the 22 targeted miRNAs had expressions that were significantly associated with survival based on patients with expression (FC < 1 or FC ≥ 1).

Patients with expression level < 1 FC had longer survival than those with ≥1 FC for miR19b (HR (95% Confidence Interval (CI)), 2.5 (1.17–5.2), *p* = 0.014; median survival 30.1 vs. 21.3 months; Figure 6a) and miR-130b* (HR (95% CI), 5.4 (1.29–22.67) *p* = 0.001; median survival 50.2 vs. 23.1 months; Figure 6b). The significant findings described here for survival related to both mRNA and miRNA expression need to be confirmed and validated in studies with a larger sample size in the future.

## 4. Discussion

The present study is the first analysis in ESCC patients to simultaneously examine alterations of *CSMD1* somatic DNA in conjunction with both mRNA and targeted miRNA expression in the same patients and samples. Our results show complex patterns among alterations of DNA and expression of RNA and target miRNAs related to this gene. We identified *CSMD1* DNA alterations in two-thirds of ESCC patients. When we divided patients by the presence/absence of these alterations to further examine relations with mRNA and miRNA expression, our results indicated that *CSMD1* exhibited substantial heterogeneity in tumor nucleic acid alterations and expression levels, even though the cases all arose from the same high-risk geographic area and might be expected to show more molecular homogeneity. Recently, scientists have shown that investigating tumor heterogeneity may help us to understand the biology of tumors [30].

Somatic DNA alterations were identified in 70% of ESCC cases in this study, a higher prevalence than has been reported in other cancers, such as breast (55%) or lung cancer (46%) [5]. These differences suggest that ESCC patients may have higher instability of *CSMD1* or, alternatively, that the diverse methods used across these studies (e.g., SNP vs. CGH arrays) may have influenced these variations [6]. We also determined that most SNPs with alterations in *CSMD1* were in introns adjacent to exons 4–11, 24–25, 39–40, 55–56, and 69–70, an observation that may facilitate detecting potentially meaningful SNPs to examine in related genes. Related previous studies showed that *CSMD1* had biallelic instability and somatic mutations in exons in Chinese ESCC patients [17,18,19]. Collectively, the results suggest that *CSMD1* plays a role in the development of ESCC, although the patterns of somatic CNA, AI, LOH, and mutations appear complex and highly heterogeneous.

A major challenge in human genetics is pinpointing which non-coding genetic variants affect gene expression and influence disease risk. Since all the SNPs that showed somatic alterations in our study were located in non-coding regions of *CSMD1*, we had a similar question, namely, did the non-coding SNPs with DNA alterations we identified influence gene expression in *CSMD1*? To answer this question, we performed qRT-PCR with 11 probes, covering 16 coding regions and splice sites to capture expression in the DNA-alteration regions. In brief, we observed three phenomena: First, we found that 69% of ESCC patients with somatic DNA alterations had abnormal expression of the *CSMD1* gene. Second, we found consistency between expression of mRNA and somatic DNA alterations in 31% of cases (e.g., case E0844, overexpression and CN gain; case E1210, underexpression and CN loss), but not in others (e.g., case E1242, CN loss but overexpression (5.82 FC); case E1862, CN gain but underexpression (0.24 FC)). This phenomenon may result from several factors, such as mutations, structural variants, or methylation. Third, we found that *CSMD1* expression levels varied across different coding regions. For example, in case E0387 (with CN loss and AI), two probes were underexpressed (FCs 0.32 for exons 7–8 and 0.35 for exons 6–7), two probes (for exons 10–11 and 24–25) showed average expression, and six probes were overexpressed (2.20–9.16 FC) for other exons. Overall, expression in this case averaged 2.77 FC, but it ranged from 0.32 to 9.16 FC. There are three genes at the 5′ end of *CSMD1*, including two pseudogenes (*RSL24D1P7*, ribosomal L24 domain containing 1 Pseudogene 7; and *RN7SL318P*, RNA 7SL Cytoplasmic 318 Pseudogene), and one uncharacterized LOC107986907 (an RNA gene affiliated with the ncRNA class). It is unknown whether these genes influence *CSMD1* expression. Richter et al. reported that aberrant splicing can cause the alteration of *CSMD1* expression in cancer cell lines and may change *CSMD1* via formation of a nonsense codon through deletion of exons [12]. The probes used in our study are located on the boundary between two exons and cover RNA-splicing sites designed by ABI; thus, the alterations of these splicing sites in cases may affect *CSMD1* gene expression in tumors. Our results indicated that SNPs with CNAs in non-coding regions could affect gene expression, particularly since methylation may influence expression in these regions. The first exon of *CSMD1* contains a CpG island that is thought to cause underexpression of mRNA [12,13]. However, we did not observe a high frequency of DNA somatic alterations on exon 1 of *CSMD1* and so did not select probes to cover exon 1. Thus, we are unable to evaluate methylation on exon 1. Further, larger studies are needed to better understand individual gene expression and to determine whether splicing variants differentially influence function or expression. Other technologies, such as RNA-seq, might also help to better understand splicing variants of unknown significance.

Genome-wide association studies (GWASs) have emerged as powerful and successful tools to identify common SNPs associated with the risk of human diseases, including cancer. One approach to investigate the link between GWAS-identified variants and function is to look for variants that influence phenotype, for example, by comparing GWAS variants for differences in gene expression as determined by examination of expression quantitative trait loci (eQTL). Studies on genome-wide eQTLs in humans can help us to prioritize likely causal variants among the multiple SNPs within the regions identified by GWAS. However, most significant SNPs identified by GWAS are in non-coding regions of genes or in intergenic regions far from genes; thus, there remain challenges to explain the exact roles that “risk” SNPs identified by eQTL play in human diseases. One of our previous studies showed that SNPs in non-coding regions not only appeared to be potential risk biomarkers by classic eQTL, but also carried somatic CNAs that may influence gene expression in ESCC [24]. The present study further showed that DNA segments containing non-coding SNPs with somatic CNAs may influence mRNA expression in different coding regions. Somatic CNAs have been profiled in thousands of tumor samples of all cancer types, using dense SNP arrays. It has also been shown that somatic CNAs may cause genomic instability characteristic of each cancer, although mechanisms of action remain to be determined. Our results provided some clues related to whether non-coding SNPs with CNAs contribute to changes in gene expression, which may help to better understand the mechanisms and processes that lead to their emergence during tumorigenesis.

Gene-expression status is also influenced by target miRNAs. Lang et al. reported that both miR10a and miR-10b could repress *CSMD1* expression based on their genome-wide profiling of miRNAs expressed in glioblastoma stem cells and normal neural stem cells [21]. Our study showed that several miR expressions were correlated with the expression of selected coding regions of *CSMD1* in ESCC. For example, miR-130a and miR-130b were positively correlated with expression in multiple exons in *CSMD1*. The abnormal expression of miR-130b has also been reported in other cancers (e.g., deregulation of miR-130b and a suggested oncogenic role of the miR-130b/301b cluster in prostate cancer) [30]. Furthermore, we observed correlations between non-coding SNPs with somatic CNAs and the expression of *CSMD1* target miRNAs in ESCC (e.g., nine SNPs with CNAs were positively correlated with expression of miR-135b). Finally, seven SNPs with somatic alterations had a correlated expression of both *CSMD1* and its target miRs, although the number of SNPs was small. Altogether, our results suggest that somatic alterations in non-coding SNPs may affect the expression of both mRNA and miRs in ESCC.

There are several limitations in the present study, including the relatively small sample size. Thus, some of the significant associations reported here likely occurred by chance, and these findings need to be replicated and validated in future larger studies. Furthermore, the SNP arrays used here did not include SNPs in the exons of *CSMD1*. In addition, information on other types of somatic alterations (i.e., mutations, structural variants, and methylation) was not available for these cases. Future studies are also needed to help determine whether this integrated approach may lead to the identification of biological markers that may be used to determine clinical relevance or other mechanisms of action between *CSMD1* and ESCC.

## 5. Conclusions

In summary, our evaluation of *CSMD1* identified somatic DNA alterations and abnormal expressions of mRNA and its target miRNAs in two-thirds of ESCC patients. We also found that alterations in SNPs in non-coding regions of *CSMD1* influenced the expression of both *CSMD1* and its target miRs.

## Figures and Tables

**Table 1:** Summary of somatic alterations and expressions of mRNA and target miRNAs on *CSMD1* in 56 ESCC cases.

No	Case ID	DNA Somatic Alterations on *CSMD1*	Gene Expression (FC) ^1^	Target miRNAs (FC) ^2^
			**on** ***CSMD1***	**on** ***CSMD1***
1	E1450	Allelic Imbalance	3.67	1.77
2	E1535	Allelic Imbalance	0.87	1.41
3	E1566	Allelic Imbalance	25.25	26.53
4	E1860	Allelic Imbalance	0.36	2.07
5	E1866	Allelic Imbalance	NA	1
6	E1885	Allelic Imbalance	41.10	72.57
7	E0362	Allelic Imbalance, CN Gain	0.98	12.02
8	E0844	Allelic Imbalance, CN Gain	48.01	15.25
9	E1293	Allelic Imbalance, CN Gain	3.26	0.81
10	E1546	Allelic Imbalance, CN Gain	0.25	2.51
11	E1635	Allelic Imbalance, CN Gain	1.52	0.56
12	E1862	Allelic Imbalance, CN Gain	0.24	6.02
13	E1874	Allelic Imbalance, CN Gain	0.38	1.61
14	E0796	Allelic Imbalance, CN Gain, CN Loss	0.20	3.08
15	E1507	Allelic Imbalance, CN Gain, CN Loss	0.7	8.61
16	E1575	Allelic Imbalance, CN Gain, CN Loss	0.77	0.44
17	E0410	Allelic Imbalance, CN Gain, LOH	1.05	10.65
18	E0387	Allelic Imbalance, CN Loss	2.77	4.36
19	E1210	Allelic Imbalance, CN Loss	0.15	5.58
20	E1242	Allelic Imbalance, CN Loss	5.18	2.85
21	E1256	Allelic Imbalance, CN Loss	10.25	1.11
22	E1520	Allelic Imbalance, CN Loss	2.08	1.4
23	E1521	Allelic Imbalance, CN Loss	5.03	1.07
24	E1532	Allelic Imbalance, CN Loss	0.27	3.15
25	E1558	Allelic Imbalance, CN Loss	18.69	7.58
26	E1572	Allelic Imbalance, CN Loss	9.38	3.75
27	E1756	Allelic Imbalance, CN Loss	0.25	8.6
28	E1782	Allelic Imbalance, CN Loss	1.63	9.28
29	E1793	Allelic Imbalance, CN Loss	NA	3.34
30	E1879	Allelic Imbalance, CN Loss	59.56	1.65
31	E1897	Allelic Imbalance, CN Loss	0.42	1.83
32	E1910	Allelic Imbalance, CN Loss	1.04	1.27
33	E1542	CN Gain	186.84	13.01
34	E1584	CN Gain	7.37	9.93
35	E1416	CN Loss	7.70	2.7
36	E1510	CN Loss	0.53	7.83
37	E1610	CN Loss	1.11	38.59
38	E1475	CN Loss, LOH	6.42	8.00
39	E1864	LOH	0.52	1.47
40	E0381	not observed	1.57	3.81
41	E0742	not observed	1.65	2.35
42	E0746	not observed	3.22	1.89
43	E0822	not observed	0.45	3.00
44	E1179	not observed	47.84	25.37
45	E1195	not observed	4.81	1.55
46	E1400	not observed	1.11	7.37
47	E1415	not observed	1.25	2.8
48	E1435	not observed	0.25	2.09
49	E1451	not observed	8.54	0.76
50	E1573	not observed	0.31	0.9
51	E1589	not observed	1.62	7.6
52	E1796	not observed	0.29	1.65
53	E1877	not observed	0.72	0.71
54	E1880	not observed	5.28	6.5
55	E1882	not observed	28.97	60.39
56	E1905	not observed	2.36	2.19

^1^ FC, fold change = LOG2^signal of tumor−signal normal^, ^2^ FC, fold change = 2^−^^∆∆CT=(∆CT of tumor−^^∆CT of normal)^.

**Table 2:** The seven non-coding SNPs with somatic CN alterations were significantly correlated with both expressions of *CSMD1* and its target miR in ESCC.

			*CSMD1*				*CSMD1*	
No	rs #		mRNA Expression		rs #		Target miRNA Expression	
	(Intron)	Region	rho Value	*p* Value	(Intron)	Name	rho Value	*p* Value
1	**rs12674947 (3)**	exon 6–7	0.833	0.015	**rs12674947 (3)**	Has-miR-130b	−1.000	0.000
	**rs12674947**	exon 5–6	0.786	0.028				
	**rs12674947**	exon 4–5	0.821	0.034				
2	**rs17068837 (3)**	exon 10–11	−0.881	0.007	**rs17068837 (3)**	Has-miR-135b	0.845	0.004
3	**rs2128213 (7)**	exon 10–11	−0.821	0.034	**rs2128213 (7)**	has-miR-135b	0.879	0.000
4	**rs2554706 (3)**	exon 10–11	−0.857	0.011	**rs2554706 (3)**	has-miR-449a	−0.964	0.003
	**rs2554706**	exon 24–25	−0.648	0.049				
5	**rs7000092**	exon 69–70	1.000	0.017	**rs7000092**	has-miR-130a	1.000	0.003
6	**rs7007506 (1)**	exon 69–70	0.964	0.003	**rs7007506 (1)**	has-miR-130a	0.964	0.003
	**rs7007506**	exon 39–40	0.929	0.007				
7	**rs9693235 (3)**	exon 9–10	0.964	0.003	**rs9693235 (3)**	has-miR-454	0.905	0.005
	**rs9693235**	exon 4–5	0.786	0.048				

## Data Availability

The GEO accession number for SNP array data used here is GSE74705. The GEO accession number for miRNA expression array data used here is GSE67269.

## Supplementary Materials

The following supporting information can be downloaded at https://www.mdpi.com/article/10.3390/cancers14205001/s1. Figure S1a (case E1782): The top panel shows somatic alterations in *CSMD1* in a case based on SNPs. Blue indicates copy-number (CN) gain; red indicates CN loss; purple indicates allelic imbalance (AI). The middle panel shows circles that indicate the position and Log R ratio (LRR) value for individual SNPs. Green indicates CN gain; red indicates CN loss. The bottom panel circles indicate the position and B allele frequency (BAF) for individual heterozygous SNPs. This panel also shows AI (exons 1–2 and exons 4–5), as determined from the SNP array. Figure S1b (case E1635): The top and middle panels show a large *CSMD1* CN gain region that covers almost the whole gene divided into three regions (exons 1–3, exons 4–5, and exons 6–70); the bottom panel shows AI on the whole gene from SNP array. Figure S1c (case E1575): The top and middle panels show mixed CN alterations (both loss and gain), including one CN loss region (exons 6–70) and one CN gain region (exons 2–4); the bottom panel shows AI (exons 1–6) from SNP array. Figure S1d (Case E0387, GE003): The top and middle panels show two CN loss regions: exons 5–6 and exons 3–4. The bottom panel shows one AI region: exons 6–7. Figure S1e (Case E0410, GE059): The top and middle panels show CN gain and LOH regions from exons 24–70; the bottom panel shows AI on the same region as CN gain and LOH. Table S1: Clinical characteristics and risk factors in ESCC cases. Table S2: Probes for *CSMD1* expression in ESCC, using qRT-PCR in ESCC. Table S3: Details of somatic alterations on *CSMD1* based on SNP array in 56 ESCC cases. Table S4: *CSMD1* gene expression in ESCC cases, using qRT-PCR. Table S5: Expression of miRNA targets for *CSMD1* in 56 ESCC cases. Table S6: (a) Significant correlations between somatic CN alterations and expression of *CSMD1* in ESCC. (b) Significant correlations between somatic CN alterations and expression of *CSMD1* target mRNAs in ESCC. (c) Significant correlations between expressions of *CSMD1* and its target miRNAs in ESCC. Table S7: (a) *CSMD1* expression in ESCC patients with and without somatic CN alterations, AI, and LOH. (b) Expression of miRNA targets for *CSMD1* in ESCC patients with and without somatic CN alterations, AI, and LOH.
