# Supplementary figures and images for: CSMD1 Shows Complex Patterns of Somatic Copy Number Alterations and Expressions of mRNAs and Target Micro RNAs in Esophageal Squamous Cell Carcinoma
Source: Cancers (Basel). 2022 Oct 13;14(20):5001. doi: 10.3390/cancers14205001 (PMC9599939; doi:10.3390/cancers14205001)

p23.2

Zygosity

Genes

Exons

CNVs

miRNA

KIAA1890

CSMD1

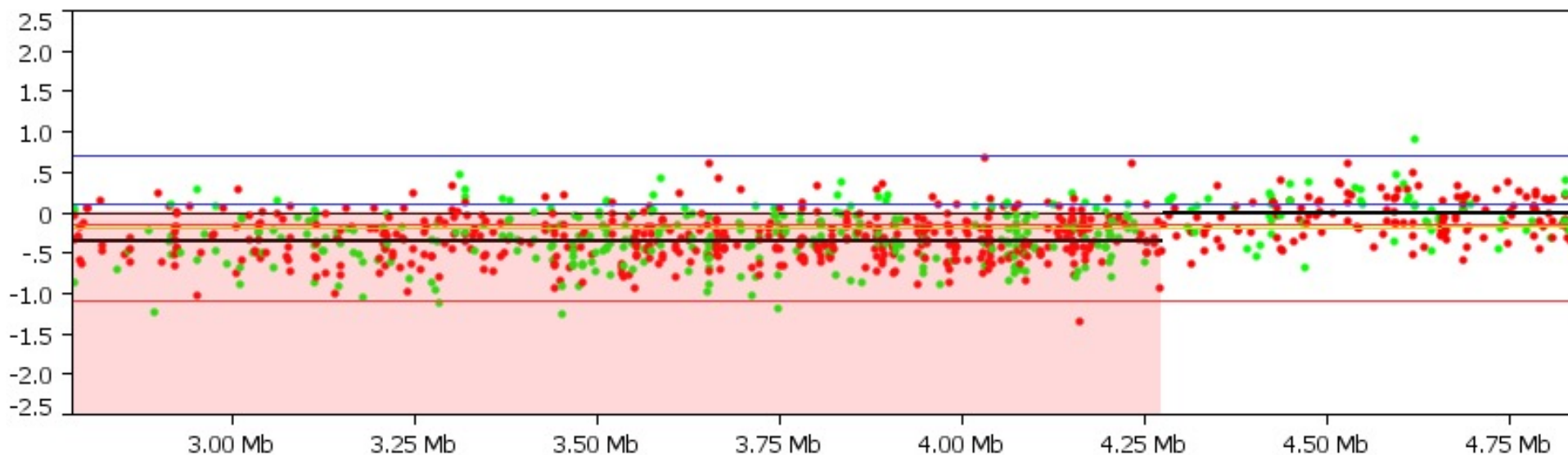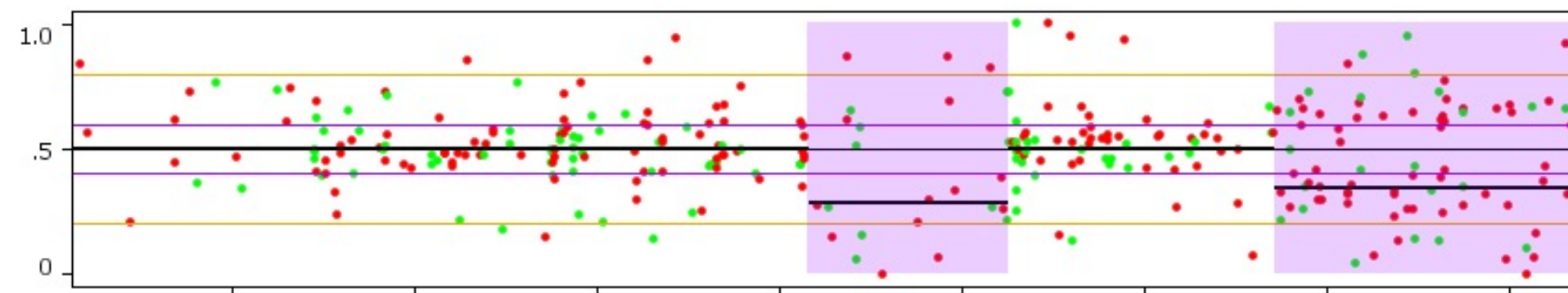

E1782: Chromosome 8

Supplement: Supplementary file 1 [file cancers-14-05001-s001.zip › S Fig 1a E1782 CN AI CSMD1 SNP array Feb 14 2022 v2.pdf]

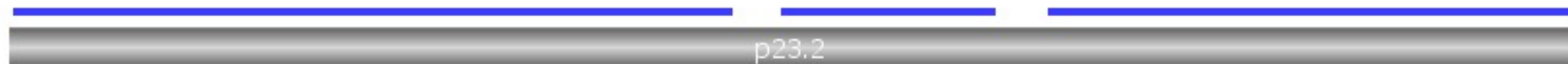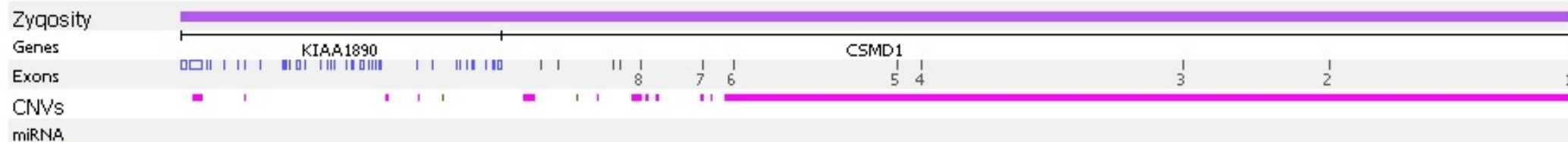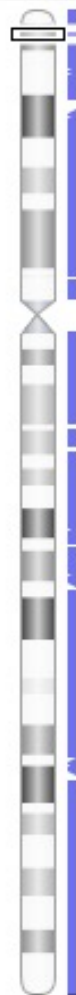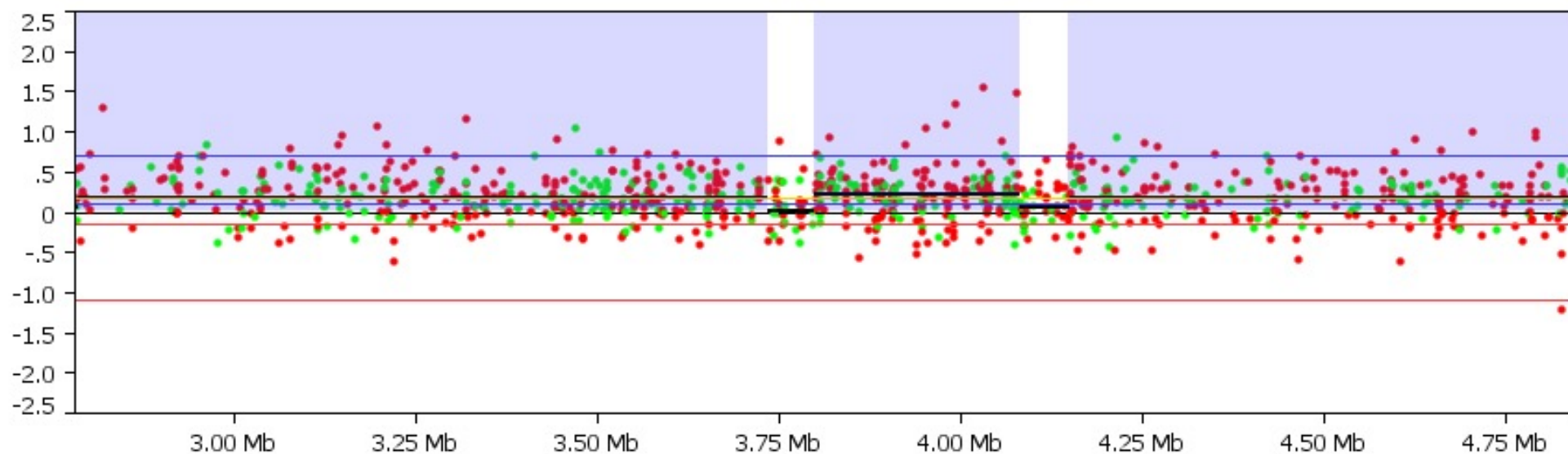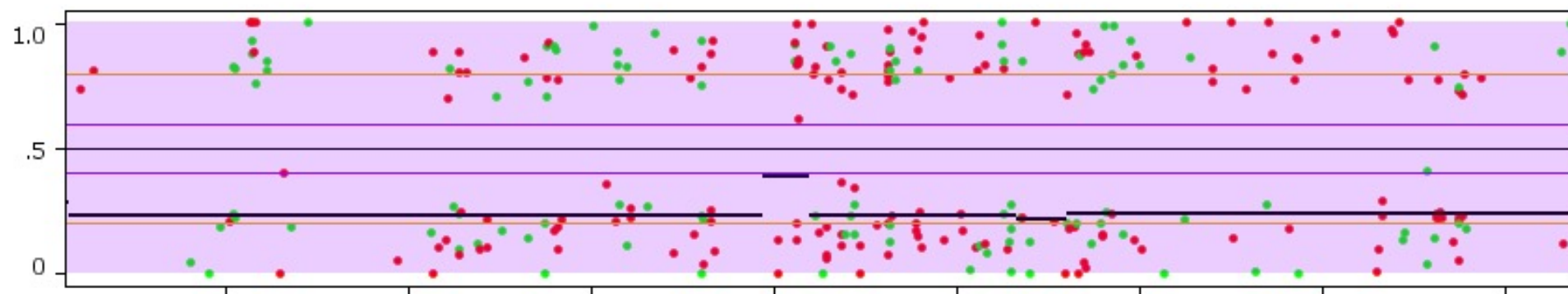

Supplement: Supplementary file 1 [file cancers-14-05001-s001.zip › S Fig 1b E1635 CN AI CSMD1 SNP array Feb 14 2022 v2.pdf]

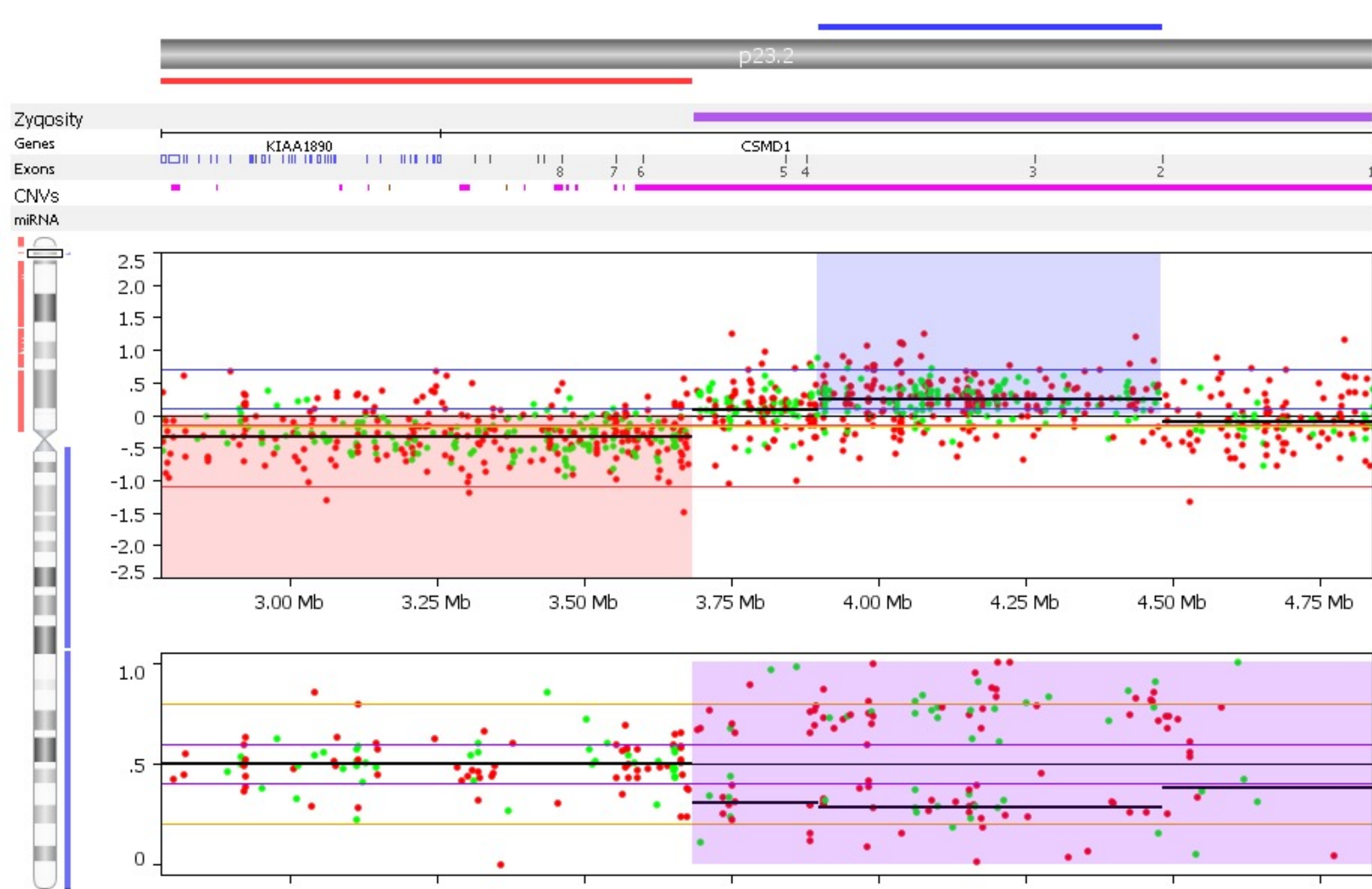

E1575: Chromosome 8

Supplement: Supplementary file 1 [file cancers-14-05001-s001.zip › S Fig 1c CN AI on E1575 CSMD1 on SNP array Feb 14 2022 v2.pdf]

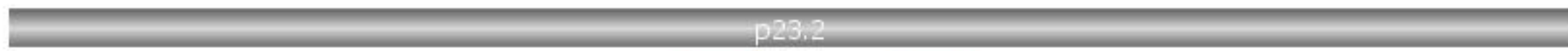

Zygosity

Genes

Exons

CNVs

miRNA

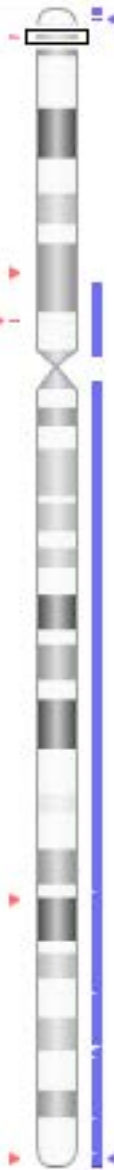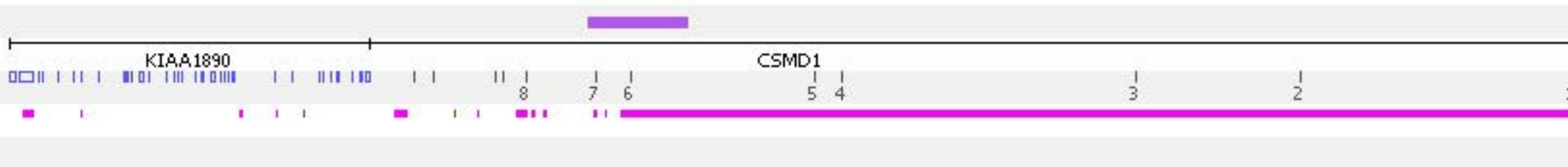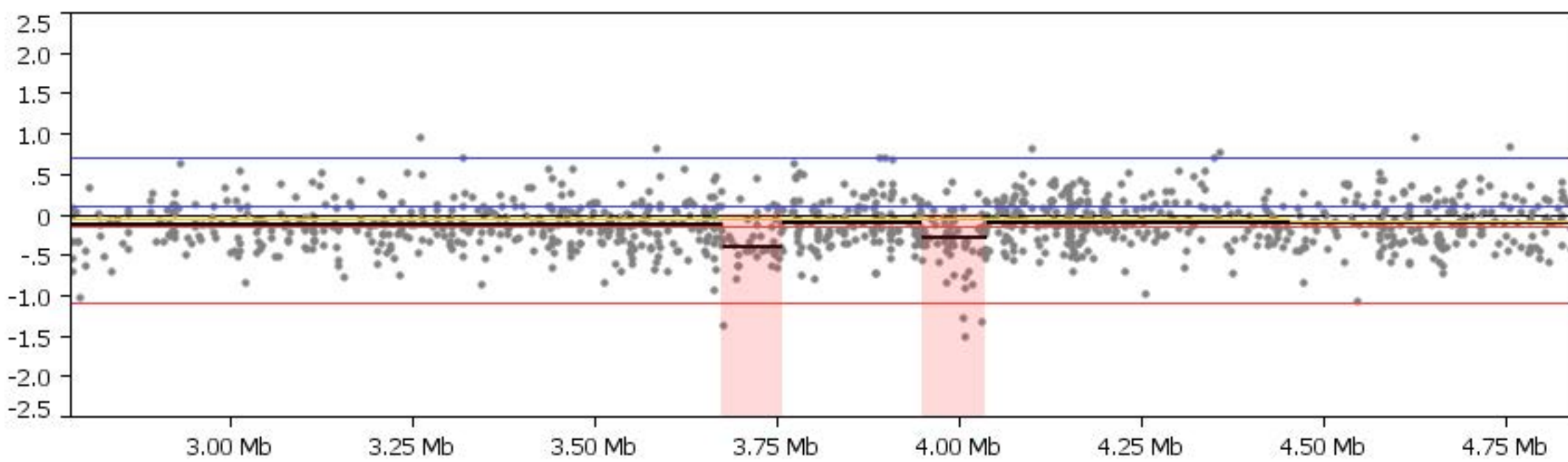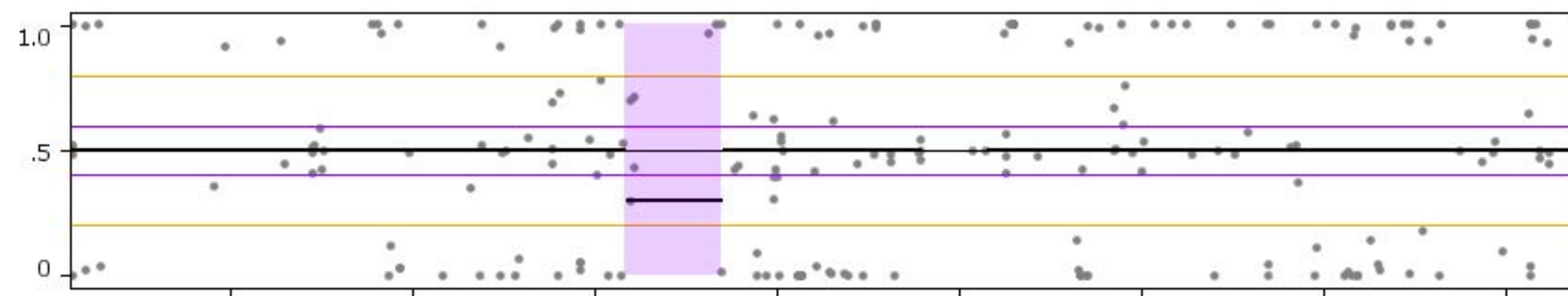

GE003: Chromosome 8

Supplement: Supplementary file 1 [file cancers-14-05001-s001.zip › S Fig 1d E0387 GE003 CSMD1 SNP array Oct 2022.pdf]

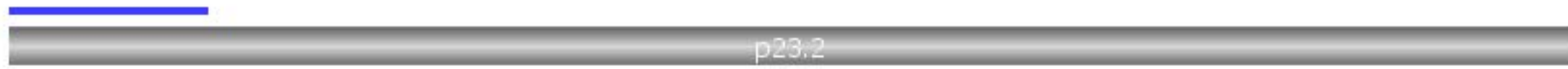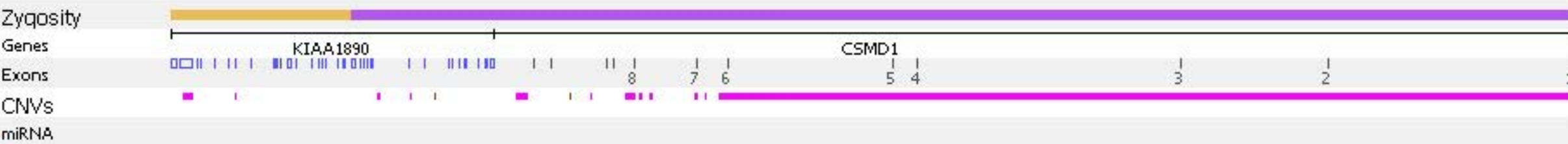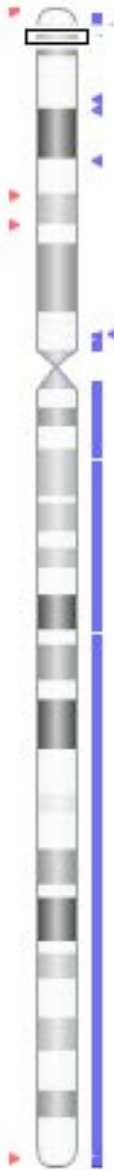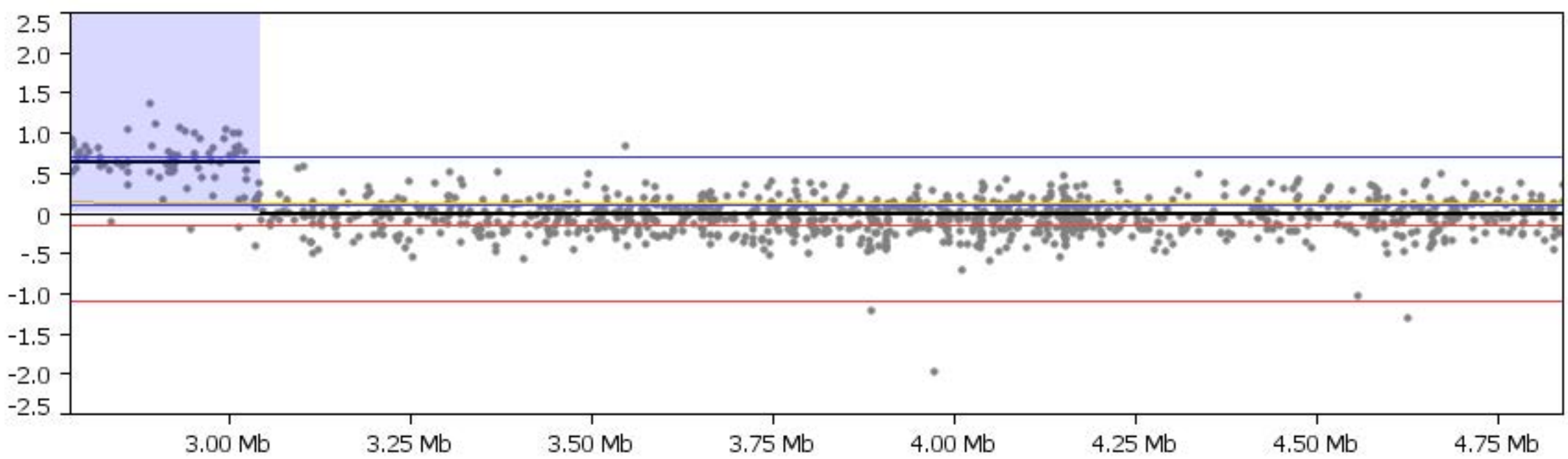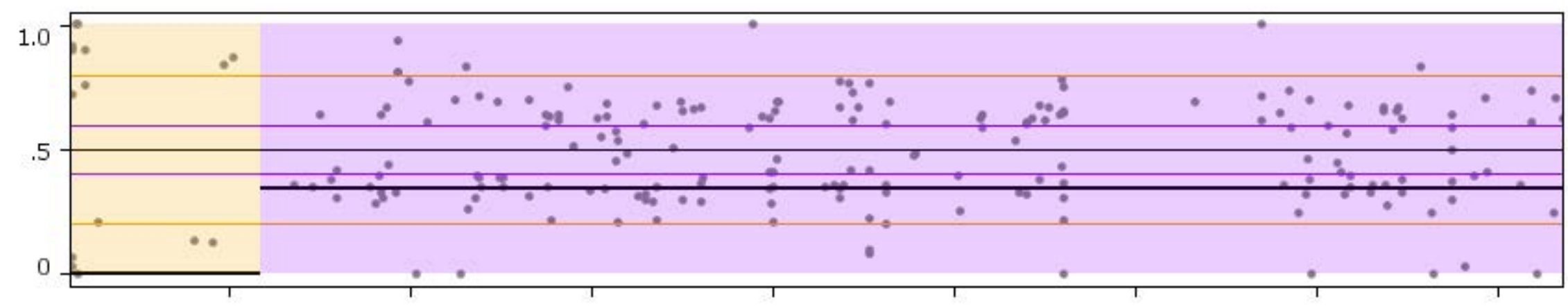

GE059: Chromosome 8

Supplement: Supplementary file 1 [file cancers-14-05001-s001.zip › S Fig 1e E0410 GE059 CSMD1 SNP array Oct 2022.pdf]
